# Screening for Cervical Cancer: A Comprehensive Review of Guidelines

**DOI:** 10.3390/cancers17132072

**Published:** 2025-06-20

**Authors:** Evgenia Zampaoglou, Eirini Boureka, Evdoxia Gounari, Polyxeni-Natalia Liasidi, Ioannis Kalogiannidis, Zoi Tsimtsiou, Anna-Bettina Haidich, Ioannis Tsakiridis, Themistoklis Dagklis

**Affiliations:** 1 Department of Hygiene, Social & Preventive Medicine and Medical Statistics, School of Medicine, Faculty of Health Sciences, Aristotle University of Thessaloniki, 541 24 Thessaloniki, Greece; zlevgeni@past.auth.gr (E.Z.); evigounari@auth.gr (E.G.); ztsimtsiou@auth.gr (Z.T.); haidich@auth.gr (A.-B.H.); 2 Third Department of Obstetrics and Gynecology, School of Medicine, Faculty of Health Sciences, Aristotle University of Thessaloniki, 541 24 Thessaloniki, Greece; ebourek@auth.gr (E.B.); pliasi@auth.gr (P.-N.L.); ikalogia@auth.gr (I.K.); dagklis@auth.gr (T.D.)

**Keywords:** cervical cancer, cytology, HPV testing, prevention, screening, guidelines, comparison

## Abstract

Cervical cancer remains one of the main causes of female mortality, especially in middle- and low-income countries, despite efforts towards the implementation of global vaccination against human papillomavirus (HPV). The aim of this study was to review and compare the most recently published international guidelines providing recommendations on cervical cancer screening strategies among average and high-risk women. Thus, a comparative review of guidelines by the US Preventive Services Task Force (USPSTF), the American Cancer Society (ACS), the American Society of Clinical Oncology (ASCO), the World Health Organization (WHO), the Canadian Task Force on Preventive Health Care (CTFPHC), the Cancer Council Australia (CCA), and the European Guidelines (EG) was conducted. There is an overall agreement regarding the suggestions made for women younger than 21 and those older than 65, with all guidelines stating against routine screening, with the exceptions of CTFPHC and CCA that expand the age group to up to 70 and 75 years, respectively. Continuation of screening in older women is also suggested in those with a history of a precancerous lesion and those with inadequate screening. Most guidelines recommend routine screening at 30–65 years, while the WHO advises that screening should be prioritized at 30–49 years. HPV DNA testing is the method of choice recommended by most guidelines, followed by cytology as an alternative, except for CTFPHC, which refers to cytology only, with self-sampling being an acceptable method by most medical societies. Agreements exist regarding recommendations for specific groups, such as women with a history of total hysterectomy for benign reasons, women with a complete vaccination against HPV, individuals from the lesbian, gay, bisexual, transgender, and queer communities and women with multiple sexual partners or early initiation of sexual activity. On the other hand, the age group of 21–29 is addressed differently by the reviewed guidelines, while differentiations also occur in the screening strategies in cases of abnormal screening results, in women with immunodeficiency, those with in utero exposure to diethylstilbestrole and pregnant women. The development of consistent practice protocols for the most appropriate cervical cancer screening programs seems to be of major importance to reduce mortality rates and safely guide everyday clinical practice.

## Simple Summary

Cervical cancer screening programs vary across the globe. This descriptive review aimed to compare the recommendations provided by the most influential guidelines on cervical cancer screening. Thus, it may be easier for busy clinicians to make relevant decisions. Following a comparison, we identified that most of the reviewed guidelines agree that cervical cancer screening is not recommended for women younger than 21 and those older than 65. Moreover, the majority of the guidelines recommend initiation of screening at 30 years, and HPV DNA is probably the appropriate method. Of note, homophony exists regarding recommendations for women with a history of a total hysterectomy for benign reasons, those with complete vaccination against HPV and women with multiple sexual partners. On the other hand, discrepancies exist in the screening strategies in cases of abnormal screening results and pregnant women. The development of consistent guidelines based on local vaccination coverage seems of major importance to minimize cervical cancer mortality rates.

## 1. Introduction

Cervical cancer remains one of the leading causes of female mortality globally, affecting almost half a million women every year [1]. While the overall mortality rate has significantly decreased in high-income countries during the last decades, it remains high in middle- and low-income countries, with 88% of deaths occurring in these settings [2].

The most prevalent risk factors for cervical cancer include infection with human papillomavirus (HPV) [3], especially certain high-risk subtypes, infection with Human Immunodeficiency Virus (HIV) or other causes of immunosuppression [4], in utero exposure to diethylstilbestrol (DES) [5] and a previous history of precancerous cervical lesions, such as cervical intraepithelial neoplasia (CIN) grade 2 or 3 [6]. Women presenting with these characteristics require close surveillance with more frequent screening compared to the general population to minimize mortality rates. Moreover, women of low socioeconomic status or from rural areas are more likely to miss screening appointments, increasing the likelihood of a cancer diagnosis in the future [2].

Screening for cervical cancer aims primarily at the detection of precancerous lesions to prevent their progression to invasive disease; cytology was the most commonly used method, either in the form of conventional (Pap smear) or liquid-based [7], in order to identify cervical pathology, while HPV DNA testing has been recently introduced, especially in high resource settings, either alone or in combination with cytology [8]. Additionally, visual inspection with acetic acid (VIA) is commonly used in low-income countries, but it demonstrates lower specificity than other methods [9].

The development and implementation of effective vaccines against high-risk HPV subtypes, especially 16 and 18, have significantly reduced HPV-related precancerous lesions [10]. However, while this is a highly effective strategy to drastically reduce the incidence of cervical cancer, implementation still remains at a suboptimal level, especially in low-income countries [11]. Similarly, the implementation of screening strategies has not yet been adopted by all settings and populations, creating barriers to the reduction of mortality from cervical pathology.

These facts underline the importance of adequate cervical cancer screening based on the development and implementation of uniform international evidence-based algorithms to safely guide healthcare providers toward reducing the incidence of malignant lesions and the associated mortality rate. Thus, the aim of this descriptive review was to summarize and compare the most recent recommendations from influential guidelines on cervical cancer screening.

## 2. Evidence Acquisition

The most recently published guidelines on screening for cervical cancer were retrieved, and a descriptive review was conducted. In particular, seven guidelines were identified from the US Preventive Services Task Force (USPSTF 2018) [12], the American Cancer Society (ACS 2020) [13], the American Society of Clinical Oncology (ASCO 2022) [14], the World Health Organization (WHO 2021) [15], the Canadian Task Force on Preventive Health Care (CTFPHC 2013) [16], the Cancer Council Australia (CCA 2022), [17] and the European Guidelines (EG 2015) [18].

An overview of recommendations is presented in Table 1 (recommendations for average-risk women) and Table 2 (recommendations for high-risk and specific populations).

## 3. Screening Methods

Several diagnostic tools are used as screening methods for cervical cancer, including cytology, either conventional or liquid-based, HPV DNA testing, a combination of the previous (co-testing) or VIA. The reviewed guidelines present discrepancies regarding the most appropriate screening method. More specifically, USPSTF, ACS and CCA favor either cytology, HPV DNA testing or co-testing, EG supports either HPV DNA testing or cytology, while ASCO supports HPV testing or VIA, and the WHO accepts every method depending on each setting. Data from a large cohort study showed that HPV DNA testing presented with a higher detection rate of CIN3+ lesions compared to cytology (OR: 1.28; 95% CI: 1.2–1.6) but resulted in more colposcopy referrals (9.8% vs. 7.8%, respectively) [19]. Moreover, results from another cohort showed that co-testing presents with higher sensitivity but lower specificity compared to cytology or HPV testing alone [20]. More specifically, in 2627 screened women, cytological sensitivity (47%) was lower than HPV (95%) for CIN2+. Co-testing demonstrated higher sensitivity (99%) but at the cost of lower specificities (92–95%) compared with HPV stand-alone (95%) and cytology (97%) [20]. As a result, each of the above-mentioned methods may serve as an effective screening method for cervical cancer. However, EG underlines the downsides of co-testing, mentioning that the increased costs, higher referral rates, and insignificant increase of sensitivity compared to primary HPV testing make this method non-viable and therefore recommends against its use.

On the other hand, VIA is a method with significantly lower specificity (58.6%; 95% CI: 56.7–60.5) compared to HPV DNA testing [9] but is more suitable in low-resource settings due to its low cost. Therefore, since ASCO and the WHO are the only medical societies referring to basic settings, their recommendation is justified. However, the WHO points out that VIA is not a suitable method for women without a visible transformation zone; therefore, screening with this method should be carefully implemented. Of note, CTFPHC recommends cytology alone as a screening method due to the assumption that HPV DNA testing lacks adequate data to support its routine use. Notably, this specific guideline was published more than a decade ago.

Moreover, ASCO, the WHO, and the CCA recommend self-sampling as a safe alternative while additionally highlighting the cost-effectiveness and feasibility of such a policy. On the other hand, ACS is against this strategy as this screening method is not approved by the Food and Drug Administration (FDA), while USPSTF supports that further research is required before making a specific recommendation. Of note, EG accepts self-sampling as an alternative method only in population-organized settings, especially in women who have missed previous screening appointments, as results from a randomized trial showed a significantly higher rate of screening in women who received self-sampling kits (RR: 1.41; 95% CI: 1.10–1.82) [21]. For women that do not have access to or who are reluctant to undergo pelvic examination, self-collected vaginal samples can be used for HPV testing [22]. More specifically, patients can collect samples from the vagina using a tampon, cotton swab, cytobrush, or cervicovaginal lavage; self-collection can be performed under supervision at a clinic or at home [22]. However, EG implies that this method should not be preferred over clinician-collected sampling, as data show that its sensitivity is significantly lower than clinician-collected specimens (76% vs. 91%) [23]. Of note, in a meta-analysis including 56 accuracy studies and 25 randomized trials, self-collected compared with clinician-collected samples had similar absolute pooled sensitivity (96%) when high-risk HPV (hrHPV) assays for CIN2+ based on polymerase chain reaction were used [24]. In addition, patients receiving a self-sampling kit rather than a reminder letter to attend a clinician-screening appointment had higher response rates (pooled relative participation rate: 2.3%; 95% CI: 1.9–2.9); however, response rates were highly variable among different settings [24]. 

Interestingly, EG is the only medical society referring to specific strategies to increase screening attendance and compliance. More specifically, invitation letters, reminder letters and telephone reminders have been shown to significantly increase compliance rate, with a 1.5-fold higher compliance rate in women receiving a letter compared to standard care on no reminder [25]. Moreover, telephone invitations resulted in successful screening more efficiently than by letter (RR: 1.32; 95% CI: 1.15–1.53) [25]. Additionally, EG suggests minimizing if not entirely eliminating, the financial cost of screening, resulting from a systematic review indicating that reducing costs was the second most effective strategy for increasing screening rates [26].

## 4. Screening Intervals

There is an overall agreement regarding the optimal screening intervals for performing diagnostic methods for cervical cancer screening. More specifically, all the reviewed guidelines suggest cervical cytology in 3-year intervals, except for ASCO, which does not support this specific screening method. This is based on a large study conducted for the USPSTF, which showed that cytology screening every year or every three years resulted in similar life-years gained (69,248 vs. 69,213, respectively) [27]. Also, the number of false positive results and colposcopy referrals were significantly higher in the annual strategy. On the other hand, results from a cohort study showed that when comparing 3- to 5-year intervals, there was a higher incidence of cancer in women with screening every 3.5 to 5.5 years than in those with screening every 0.5 to 3.5 years (RR: 2.2; 95% CI: 1.3–3.8) [28].

Moreover, an agreement exists regarding HPV DNA testing with all medical societies, except CTFPHC, that refers to cytology only, agreeing on 5-year intervals. Of note, 5-year intervals are suggested for co-testing, as well, by USPSTF and ACS. Notably, the recommendation from ASCO applies only to maximal and enhanced settings. Similarly, with cytology, results from a study showed that when comparing 3-year and 5-year HPV screening strategies, although equal numbers of life-years were gained on both occasions, a shorter interval resulted in more unnecessary procedures and tests [29]. Additionally, the WHO compared 5- to 10-year intervals and concluded that in screening every 5 years, a 57% reduction of cancer cases would occur, compared to only 35% in 10-year intervals [30]. As a result, the 5-year interval on HPV DNA screening appears to be the most beneficial, not only in terms of cancer cases prevented and unnecessary procedures reduced, but also in terms of overall cost-effectiveness. On the other hand, EG mentions that longer than 5-year intervals could be applicable, provided that previous history is carefully evaluated and adequate screening is ensured. This is based on studies indicating that co-testing 6–10 years after negative HPV DNA resulted in lower CIN3+ incidence compared to standard 3-year cytology screening [31,32].

Additionally, as already mentioned, VIA is suggested only by ASCO and the WHO for low-income countries and settings where more costly methods are not applicable. More specifically, ASCO suggests screening every 5–10 years, whereas the WHO recommends 3-year intervals. The WHO’s guidelines are based on data showing that 3-year intervals of screening with VIA would result in almost 40% reduction in the incidence of cervical cancer compared to only 30% in 5-year intervals [30].

## 5. Screening Recommendations for Women at Average Risk

### 5.1. Recommendations for Women Under 21 Years

There is a consensus among the reviewed guidelines regarding women aged <21 years, with all medical societies stating against routine screening. This is based on data showing that not only this type of cancer has a very low incidence in this age group [33], but precancerous lesions appear at very low rates, as well, accounting for almost 0.2% [34]. On the other hand, these lesions, if diagnosed, could lead to an increase in treatment procedures, leading to a possible rise in adverse obstetric outcomes in the future, such as preterm delivery [35]. Therefore, given the slow progression of this specific malignancy, it seems more likely to cause harm than have a benefit in women younger than 21 years old.

### 5.2. Recommendations for Women at 21–29 Years

Notably, this age group presents with several discrepancies. More specifically, all medical societies are against routine screening for women between 21 and 24 years, except for USPSTF, which suggests routine cytology examination every 3 years for all women older than 21 years, and EG is against HPV testing prior to the age of 30 but accepts cytology screening after the age of 20 (every 3 years). The recommendation on cytology as a screening method is based on data that showed that implementation of HPV DNA testing in women younger than 30 years would dramatically increase the rate of colposcopies (approximately 200 colposcopies per 1000 women screened), with no actual benefit in cancer’s reduction [36]. Moreover, given the high prevalence of HPV in this age group, a significant increase in overdiagnosis of transient infections would occur [37]; therefore, cytology is preferred over HPV DNA testing.

The reviewed medical societies (except USPSTF and EG) recommend initiation of screening after 25 years, with differentiations regarding the appropriate screening method. This recommendation is based on data showing that in cases where HPV DNA testing was implemented after 25 years, there were similar survival rates and fewer colposcopies compared to the strategy of screening with cytology after 21 years and switching to HPV DNA testing in women aged over 25 years [29]. Additionally, similarly with the <21 years-of-age group, the recommendation against routine screening <25 years protects against overdiagnosis and overtreatment that would, consequently, lead to more cervical surgical procedures and increase the incidence of preterm deliveries [35] and antenatal cervical procedures [38]. Moreover, further data supported the initiation of screening in this age group, as there were almost 15% more cancers prevented and 7% fewer deaths occurring in women who underwent screening earlier than 30 years old, proving a significant benefit from initiation of screening <30 years [29].

On the other hand, the WHO is against routine screening in women in the average risk group under the age of 30. According to data, only a small number of cancer cases will present before the age of 30, with the same applying to precancerous lesions, as well [1]. Moreover, a study conducted by the WHO showed that almost 60% of women < 30 years old with dysplasia will experience regression from CIN2 to CIN1 or less during 24 months, compared to 44% of women older than 30 [30]. This highlights the risk of unnecessary interventions in women < 30 years, which would create a significant burden on health systems globally. Notably, the WHO provides recommendations regarding countries all over the world, but especially middle- and low-income ones, with health systems unable to financially support controversial or radical approaches. Therefore, the decision to support screening after 30 years is the most balanced benefit-to-risk approach for these settings.

On the screening method, ACS, ASCO and CCA recommend HPV testing every 5 years as the first choice, followed by co-testing every 5 years and cytology every 3 years as alternatives. According to published data, HPV DNA testing presents with a significantly higher sensitivity compared to cytology on the diagnosis of CIN3+ lesions and invasive cancer [39]. More specifically, the incidence of CIN3+ in the following 3 years for women screened negative with HPV testing or cytology was 0.11% and 0.5%, respectively [31]. Differentiation is detected by CTFPHC, which recommends cervical cytology every 3 years, initiating at 25 years. As previously mentioned, this medical society refers only to cytology as an accepted screening method due to a lack of adequate data to support HPV DNA testing implementation.

ASCO makes specific recommendations based on every setting, with the categorization into maximal, enhanced, limited and basic. Notably, the recommendations for initiation of screening in women > 25 years refer only to maximal settings. ASCO is against routine screening in enhanced, limited and basic settings as cost-effectiveness analyses showed that it is more efficient to provide screening appointments in women with no previous or very limited cervical cancer screening rather than expanding screening per lifetime in women who already have access [40].

### 5.3. Recommendations for Women at 30–64 Years

For women 30–64 years of age, agreement exists among USPSTF, ACS, ASCO (only relating to the maximal and enhanced setting), CCA and EG on the recommendation of screening with HPV DNA testing every 5 years. Additionally, cytology every 3 years (USPSTF, ACS, EG) and co-testing for 5 years (USPSTF, ACS) are acceptable alternatives. This is based on a study that compared screening strategies in terms of cervical cancer deaths per 1000 women and concluded that in women > 30 years screened with HPV DNA testing at 5-year intervals, the number of deaths was calculated at 0.29 per 1000 women compared to a significantly higher 8.34 in women with no screening at all [29]. Moreover, regarding cytology screening every 3 years and co-testing every 5 years, the respective number of deaths was 0.76 and 0.30 [29]. However, as pointed out, the implementation of co-testing yielded an increased number of tests performed, leading to a significant rise in cost. Therefore, while co-testing remains an acceptable method, HPV DNA testing and cytology may be the tests of choice. Of note, ASCO accepts 10-year intervals of HPV DNA testing after two consequent negative results at 5-year intervals in enhanced settings. Similarly to the previous age group, CTFPHC supports screening with cervical cytology every 3 years. This is based on a cohort study that showed screening with a 3-year follow-up was associated with fewer new cancer cases detected annually (RR: 0.4; 95% CI: 0.2–0.6) [41]. Additionally, the rate of high-grade precancerous lesions has been decreased in women undergoing screening [42]. Moreover, this medical society mentions that any possible adverse effects of frequent screening, especially related to obstetric outcomes, are surpassed as many women will have their childbearing complete in this specific age group.

On the other hand, ASCO (in limited and basic settings) and the WHO recommend routine screening only in women between 30 and 50 years old (the WHO states that women aged 50 to 65 with no prior screening history should be offered screening if feasible). More specifically, in limited settings, ASCO recommends HPV DNA testing every 10 years, whereas in basic settings, VIA every 5 to 10 years is considered acceptable. The WHO supports HPV testing every 5 to 10 years or, as an alternative, VIA or cytology every 3 years. These recommendations are based on evidence that cervical cancer’s incidence is considered low before the age of 30 [1]. Moreover, as previously noted, a strategy to increase the number of women who participate in screening seems more rational and efficient than increasing the frequency or the years of testing for each woman [40]. Therefore, a recommendation of screening with HPV testing every 10 years, translating to two to three times per lifetime, seems both an efficient and cost-effective strategy, as data show a reduction in mortality of 34.2% in twice-per-lifetime screening strategies [43]. Notably, a switch to 5-year intervals could be offered when it could be fully supported by these settings. According to both ASCO and the WHO, VIA is considered an acceptable alternative method in low-resource settings. Of note, the WHO suggests that women aged 50 to 65 years who have never undergone previous screening should be offered screening when resources are available.

### 5.4. Recommendations for Women over 65 Years

With regards to women > 65 years, USPSTF, ACS, ASCO, the WHO, and EG are against routine screening in all women from the average risk group with adequate previous screening (USPSTF, ACS, ASCO, and EG) or regardless of screening history (WHO). This recommendation is based on data showing that women with adequate previous screening were less likely to develop cancer or precancerous lesions after the age of 65, and even if it occurred, the disease’s progression would not result in invasive cancer soon enough to reduce a patient’s life-expectancy [44,45]. According to a mathematical model, only 1.6 cancer cases were prevented per 1000 women, in women with continuous screening until the age of 90 [27]. On the other hand, continuing screening would carry more problems, considering the patient’s discomfort and potential harm from unnecessary surgical procedures. The same model concluded that in only 1 year per 1000 women, there was an extension of life expectancy while resulting in numerous unnecessary procedures and false-positive results [27]. However, as it is pointed out, adequate screening should be ensured before suggesting screening cessation. In any case, since life expectancy is increasing, more published data are needed on the specific screening methods and cessation timing for these populations.

On the other hand, CTFPHC recommends screening up to 70 years with cytology every 3 years, ASCO suggests individualization of screening up to 70 years in maximal settings, while recommendations from CCA support screening up to 74 years in women at the average risk group or continuous screening in women > 75 years (self-collected specimen or by a clinician) without previous screening. Results from a Swedish study showed that women older than 70 with invasive cancer were less likely to report previous screening compared to healthy ones (OR: 0.4; 95% CI: 0.2–0.5) [46]. Moreover, another study pointed out a possible benefit from screening up to the age of 75, as women with cancer from this age group had lower screening rates compared to individuals without cancer (32% vs. 40%, respectively) [47]. Of note, all the reviewed guidelines agree on the lack of adequate data to fully support a specific age group as the upper limit for screening, as well-designed studies did not include older women in their population.

### 5.5. Adequate Screening

All the reviewed guidelines, except from EG, refer to the term of adequate screening, especially in relation to the decision to terminate screening in the previously mentioned older age groups. As previously mentioned, USPSTF and ACS suggest the cut-off of 65 years, ASCO and CTFPHC the upper limit of 70 in individual cases and CCA the age of 75. On the other hand, the WHO recommends the earliest age for screening cessation, at 50 years, while proposing that women aged 50 to 65 with no prior screening history should be offered screening when feasible.

USPSTF, ACS and CTFPHC agree that three consecutive negative cytology results or two consecutive negative HPV results in the last ten years (USPSTF, ACS) with the last one within the last 3–5 years are considered adequate screening, while, in a less specific manner, ASCO describes negative results within the last 15 years, in maximal and enhanced settings only. CCA limits the timeframe, suggesting one negative HPV DNA testing in the last 5 years as adequate screening to safely guide towards screening cessation at 70 to 74 years. In addition, the WHO suggests screening cessation after two consecutive negative results. The existence of prior screening is extremely important in the strategy for cervical cancer prevention, as previous abnormal results or cervical pathology are among the primary risk factors for cervical cancer [6]. Moreover, data from the US have shown that while almost 90% of women aged less than 40 years of age have undergone screening, only 80% of women aged 50–65 have been reported to be regularly screened [48]. This underlines the progressive lack of adequate documentation of women’s health status, which may lead to missed opportunities for early detection and prevention. Notably, as previously mentioned, data from a case-control study showed that among cancer patients, the number of women with no screening was significantly higher [47], highlighting furthermore the need for sufficient screening.

Additionally, all medical societies, except the WHO and EG, that do not address this topic recommend screening cessation in women who undergo total hysterectomy for benign reasons without a prior history of high-grade cervical dysplasia (e.g., CIN2+ lesions). However, careful confirmation of cervical removal should be undertaken, either through a review of the medical records or by direct clinical examination of the patient. According to a large cross-sectional study, women with a previous history of total hysterectomy had a 10 times lower risk of abnormal cytology results (OR: 0.09; 95% CI: 0.02–0.24) [49]. Additionally, results from another study of almost 6000 women showed that of 9610 cytology examinations conducted, only 104 resulted in abnormal findings [50]. These findings highlight the extremely low incidence of cervical cancer in women who have undergone total hysterectomy, justifying the recommendation for screening cessation.

The same recommendations apply to women of any age group with limited life expectancy, as continued screening would be neither beneficial nor cost-effective.

## 6. Screening Recommendations for Women at High Risk

### 6.1. History of Precancerous Lesion

Screening in women with a history of CIN2+ lesions requires appropriate modalities and more careful evaluation. Relevant recommendations are provided by USPSTF, ACS, ASCO, the WHO, and CCA, with differentiations among them, while CTFPHC and EG do not refer to this topic. In detail, a more extended screening approach is suggested, with USPSTF supporting the extension of screening for 20 more years and ACS recommending screening at least 25 more years from diagnosis and surveillance every 3 years after the age of 65. Of note, CCA is in line with ACS in cases where in situ adenocarcinoma was detected and treated, where screening is suggested for 25 years, annually with co-testing and every 3 years after five consecutive negative results. Results from a cohort study showed that women with a history of high-grade dysplasia or in-situ cervical carcinoma had a significantly higher risk of developing invasive cervical cancer in the next 25 years (incidence ratio: 2.34; 95% CI: 2.18–2.50) [51]. ASCO supports screening for at least 10 years if the diagnosis was made after the age of 60, considering the parameter of life expectancy. Notably, the WHO suggests a considerably more conservative approach with repeat screening with HPV DNA testing at 12 months and, if tested negative, return to routine screening in women with CIN2/3, while in women with CIN3+ lesions, the recommended approach, following treatment, is to repeat HPV DNA every 12 months three consecutive times before returning to routine screening. This is based on an analysis conducted by the WHO, which showed that women with precancerous lesions who underwent follow-up in 12-month intervals presented with a decline in the incidence of invasive cancer, slightly lower than the 24-month follow-up strategy but with a significantly higher compliance rate [30]. In accordance with the WHO, CCA recommends screening annually with HPV DNA testing in cases where high-grade precancerous lesions were diagnosed and treated and returned to routine screening after two consecutive negative results.

### 6.2. Abnormal Results

Only the ASCO, the WHO, CCA, and EG make specific recommendations regarding the optimal strategy after abnormal primary screening results. More specifically, ASCO makes different recommendations depending on each setting, with the ones for maximal and enhanced settings following a similar approach to CCA and EG. In detail, in primary HPV DNA positive results in maximal and enhanced settings, ASCO recommends either genotyping or cytology and then repeat the HPV DNA test in 12 months if subsequent results are negative. Moreover, in cases of abnormal genotyping or cytology, the recommended approach is to directly refer the patient to colposcopy. EG is in favor of either direct colposcopy or repeat testing in 12 months in case of low-grade cytology findings, such as low-grade squamous intraepithelial lesions (LSIL) or atypical squamous cells of undetermined significance (ASCUS), as there is insufficient evidence to support either strategy more firmly. In contrast, in limited settings, following a positive HPV DNA result, ASCO recommended triage options, including cytology, genotyping, and VIA. If cytology is abnormal, colposcopy or visual assessment for treatment (VAT) is indicated. Notably, in limited settings, due to limited financial support to perform additional diagnostic tests, a positive HPV DNA test should lead directly to VAT. According to several studies, the screen-and-treat strategy proves significantly more cost-effective and helps reduce the lifetime risk of invasive cancer by almost 30%, compared to screen-triage-treat strategies [52,53], which is highly important in low-resource settings. It is highlighted that, in settings where self-collection is used, partial genotyping should be considered as it is a low-cost option and may help exclude lower-risk women from unnecessary treatment.

In accordance with ASCO’s recommendations for limited settings, the WHO suggests either genotyping, colposcopy, VIA or cytology following an abnormal HPV DNA result. However, in case of normal cytology findings, the recommended approach is to repeat HPV DNA testing in 24 months and, if the results are negative, return to the routine screening strategy. This is based on a study conducted by the WHO, which showed that following a negative triage test at 12- or 24-month intervals before the next screening is equally beneficial, but the longer period proved to be more cost-effective and resulted in fewer unnecessary procedures [30]. Of note, in cases where cytology was used as the primary screening method with abnormal results and normal colposcopic evaluation, the recommendation is to perform HPV DNA testing in 12 months and, in negative results, return to routine screening, according to data showing that 12-month follow-up after cytology triage came with more benefits compared to the 24-month interval [30]. The same study demonstrated that when comparing follow-up methods after an abnormal primary result, co-testing led to a higher number of treatments and less financial benefits than HPV DNA testing; therefore, the latter is preferred [30].

CCA differentiates itself by making recommendations based on the patient’s age. More specifically, in women younger than 50 years who test positive for HPV subtypes other than 16 or 18 but with normal cytology results, the recommended strategy is to repeat HPV DNA testing in 12 months. In the case of abnormal results, a referral for a colposcopy is recommended after three consecutive positive annual HPV DNA tests. This is based on the fact that almost 67% of these cases will resolve within a 12-month period [54], and the risk of detecting invasive cancer after a positive HPV DNA-other result is approximately 0.02% [55]. Moreover, a cost-effectiveness study demonstrated that the strategy to direct immediately to colposcopy after a positive HPV-DNA result was significantly cost-ineffective compared with a 12-month follow-up approach [56]. On the other hand, in women after the age of 50, the same approach is recommended, although a colposcopy referral is suggested after two consecutive HPV DNA tests rather than 3. Given that the clearance of HPV declines with age, the strategy to refer to colposcopy sooner in older individuals seems reasonable [54]. Of note, in cases where cytology is abnormal or HPV DNA testing results in the identification of 16 or 18 subtypes, direct colposcopic evaluation is recommended, regardless of the patient’s age. This recommendation takes into consideration the fact that subtypes 16 and 18 are associated with a high risk of future development of CIN2+ lesions and that they were present in 70% of cervical cancer cases [57]. In addition, in a modeling study conducted in Australia, this approach proved to be the most effective in reducing future cases while also offering the highest cost-effectiveness [58].

### 6.3. Inadequate Previous Screening

In women with a history of inadequate screening, USPSTF, ACS and CTFPHC agree that screening may be extended beyond the recommended age for cessation until the criteria for discontinuation are fully met. This translates to the age of 65 for USPSTF and ACS and the age of 70 for CTFPHC. Moreover, as previously mentioned, CCA supports that screening may be offered in women after the age of 75 if there was no previous adequate screening upon request of the patient. This recommendation is based on data that in older ages, most cancer cases occur in women with inadequate screening when the criteria to stop screening were not met. According to a US study, most cervical cancers diagnosed at ≥65 years occurred in women who had not met the criteria for stopping screening [59].

### 6.4. Immunodeficiency

Specific recommendations for individuals with immunodeficiency are offered only by ASCO, the WHO, CCA and EG, with the WHO providing detailed information for women living with HIV infection. ASCO does not make specific recommendations. Instead, this medical society suggests more frequent screening for immunodeficient individuals, recommending twice the number of screenings compared to the general population.

Regarding individuals living with HIV, the WHO recommends screening between the ages of 25 and 59 with HPV DNA every 3 to 5 years, preferably, or alternatively, either cytology or VIA every 3 years. This is based on data that demonstrated a higher benefit with fewer adverse obstetric and financial outcomes with HPV testing in intervals of 3 to 5 years compared to longer ones [30]. Furthermore, when comparing 3-year cytology screening with 5-year HPV screening, the overall benefit was found to be similar, but the HPV screening strategy was associated with significantly lower costs [30]. In cases with positive HPV DNA testing, triage with genotyping, colposcopy, VIA, or cytology is recommended, similar to the general population. In negative triage results, a repeat of HPV DNA testing is recommended in 12 months, compared to the 24-month interval for the general population and in consecutive negative results, return to routine screening. This is justified by a meta-analysis which showed that HIV patients had a significantly higher risk of cervical cancer development compared to the general population, and therefore, a frequent screening strategy is more appropriate [4]. Moreover, when comparing 12-month and 24-month intervals, similar harms but more benefits were found in the 12-month period [30]. Moreover, as in the general population, if primary cytology results are abnormal but with normal colposcopic findings, HPV DNA testing in 12 months is recommended and then return to routine screening in case of negative results. However, if a patient was treated for a CIN2+ lesion, two consecutive negative HPV DNA results are needed, in 12-month intervals, before returning to routine screening, as data showed that the HPV DNA test was more sensitive and resulted in fewer harms compared to co-testing or cytology alone [30]. Notably, EG makes more vague recommendations, with an initial screening strategy of twice a year during the first year of HIV diagnosis and then subsequent annual screening. Additionally, this medical society is in favor of direct colposcopic referral in abnormal cytology results, as data show that even mild cytologic atypia is frequently associated with cervical dysplasia in HIV patients [60].

As for CCA, this medical society recommends screening in 3-year intervals between the ages of 25 and 74 years, although in cases of immunodeficiency at a young age, a single test between the ages of 20 and 24 is acceptable. This is justified due to a higher risk of cervical cancer and high-grade precancerous lesions in patients with HIV infection or other conditions causing immunodeficiency [61]. Data from a cohort study showed that among transplant patients, the overall incidence of cancer was three times higher compared to non-transplant patients [62]. Moreover, it is underlined that prior to organ transplantation, careful evaluation of the patient’s health should be conducted, including cervical screening status, as patients in line for organ transplantation will receive high doses of immunosuppressive therapy after transplantation. Therefore, careful assessment of their overall health is essential to ensure that any necessary treatment is provided prior to initiation of immunosuppression. Regarding the appropriate management of abnormal results, CCA recommends a direct colposcopy in cases of positive HPV DNA results.

### 6.5. In-Utero Exposure to Diethylstilbestrol

One significant risk factor for the development of cervical cancer is in-utero exposure to DES; therefore, these women are considered high-risk. ACS and CCA are the only medical societies referring to this topic. More specifically, ACS recommends careful pelvic examination with visualization of the vaginal canal and cervix, screening with annual cytology and consideration of colposcopy. This is based on the fact that women with in-utero exposure to DES present with a significantly high risk for the development of clear cell carcinoma at young ages, usually before the age of 30 [63]. Therefore, an annual screening strategy for these women seems reasonable and should be encouraged by healthcare providers [64]. The recommendations provided by CCA support annual co-testing and colposcopy indefinitely in women with in-utero exposure to DES, based on the high incidence of cervical cancer in this population and the fact that the number of women receiving DES in Australia is unknown.

## 7. Screening Recommendations for Specific Populations

### 7.1. HPV Vaccination

There is an overall agreement among the reviewed guidelines, except the WHO and CCA, that do not make any relevant recommendations that in women who have completed the vaccination against HPV, the same age-based screening strategy should apply as in unvaccinated individuals. This is justified by the current lack of global vaccination, which limits the availability of data that can fully support a different strategy. However, it is noted that with future research, new strategies will be recommended and followed in women with immunization against this virus.

### 7.2. Pregnancy

Only CCA makes recommendations regarding pregnancy and the puerperium, while ASCO makes recommendations for the postpartum period only. In detail, during the antenatal period, CCA recommends the same strategy as in the general population, especially when screening is due or overdue, either with self-collected or clinician-collected swabs. However, the use of an endocervical brush is not recommended as excessive bleeding might occur, causing discomfort and stress to the patient. As an alternative, to-broom is the recommended tool for collecting cervical swabs. Moreover, it is highlighted that pregnancy is an excellent occasion in a woman’s life in which her overall health status can be assessed, during which cervical screening should not be overlooked.

Additionally, in cases where a positive HPV test occurs, with normal cytology results, CCA recommends repeat screening in 12 months with an HPV DNA test; in cases where subtypes 16 or 18 are identified, immediate colposcopy is recommended. In cases where cytology becomes abnormal, either with atypical squamous cells cannot rule out high-grade squamous intra-epithelial lesion (ASC-H) or high-grade squamous intraepithelial lesion (HSIL) results or glandular abnormalities, immediate colposcopy referral is essential, without waiting for the postpartum period. While pregnancy is a period where conservative management is recommended, even in high-grade lesions, colposcopy is essential for careful evaluation and differentiation between cervical dysplasia and invasive cancer [65,66]. Of note, it is underlined that colposcopic evaluation should be performed by an experienced physician in pregnancy, as physiological cervical changes occurring antenatally can often complicate accurate diagnosis [67].

Regarding the postpartum period, CCA suggests waiting at least 6 weeks before screening initiation, ideally 3 months after delivery, while ASCO supports screening either at 6 months postpartum in maximal, enhanced and limited settings or in 6 weeks in basic settings. The longer time interval is suggested to avoid unsatisfactory cytology or difficulties in interpretation of the results while regarding the 6-week recommendation, ASCO does not provide any specific justification; it is recommended as experts’ opinion. Moreover, in breastfeeding women who use vaginal estrogen supplementation, CCA suggests the use of these regimens daily prior to colposcopy, with cessation 2–3 days before the examination [68].

### 7.3. Sexual Activity

With regards to sexual activity and the age of its initiation, USPSTF, ACS and CTFPHC are in favor of applying the same screening strategies as in the general population, depending on the age group. This is recommended due to a lack of evidence suggesting that women with multiple sexual partners are at significantly higher risk of cervical cancer and should, therefore, receive more frequent screening. On the other hand, CCA mentions that in women who are sexually active before the age of 14 or those who have experienced sexual abuse, screening once before the age of 25 may be offered. This is based on the fact that HPV infection often occurs after the first sexual intercourse, and given the early age [69], potential persistent HPV infections can evolve into invasive cancer [70].

There is an overall agreement among the reviewed guidelines in relation to the lesbian, gay, bisexual, transgender, and queer (LGBTQ) community. More specifically, all medical societies recommend the implementation of the same screening strategy in all individuals with a cervix, regardless of their sexual behavior or orientation.

### 7.4. Other Conditions

CCA is the only medical society referring to women with signs and symptoms indicative of cervical cancer, such as vaginal bleeding [71]. This medical society is in favor of clinical examination, co-testing, and colposcopy, regardless of the patient’s age and the presence of blood. Vaginal bleeding is not commonly caused by cervical cancer, but it is a symptom that requires careful evaluation and investigation [72]. Moreover, results from a systematic review showed that the rate of postcoital bleeding in patients with cervical cancer ranged from 0.7% to 39%, underlining the severity of careful evaluation of any case of abnormal vaginal bleeding [73]. Additionally, as the presence of blood limits the sensitivity of both HPV tests and cytology separately, CCA recommends a combination of the methods to achieve the highest possible results. Moreover, in cases where cytology or HPV DNA test results are unsatisfactory or invalid, CCA suggests a repeat of the test immediately in case of HPV DNA test or after a 6-week period in case of cytology. Additionally, in the case of cytology, if the cause of an invalid sample is identified, efforts should be made to address the underlying issue prior to repeating the test.

## 8. Conclusions

To summarize, there is a consensus among the reviewed medical societies regarding the suggestions made for women prior to the age of 21 and after the age of 65, with all guidelines stating against routine screening, except from CTFPHC and CCA that, slightly expand the age group to up to 70 and 75 years, respectively. Expansion of the age group is also recommended in cases with a history of precancerous lesions and in cases where inadequate screening had preceded, with all the guidelines referring to the topic, recommending screening until criteria for cessation are met. Moreover, similarities are detected among the ages of 30 to 65, with an exception from the WHO that suggests 50 years as the upper limit for routine screening. Differentiations occur only regarding the appropriate screening method, with CTFPHC suggesting primary cytology as the recommended method, while all other societies recommend HPV DNA testing when applicable. Additionally, most guidelines agree regarding the term of adequate screening, with the suggestion of two to three consecutive prior negative tests within the last ten to fifteen years. CCA is an exception, which considers a single negative result within the last five years sufficient to recommend screening cessation. Agreement also exists in relation to recommendations for specific groups, such as women with a history of total hysterectomy for benign reasons, women with complete vaccination against HPV, individuals from the LGBTQ community and women with multiple sexual partners or early initiation of sexual activity. Of note, self-sampling is recommended as an alternative method by all reviewed guidelines referring to the topic, except for ACS, which advises against it due to the lack of FDA approval; USPSTF, which claims insufficient supporting data, and EG, which accepts it as an alternative in specific situations.

Several discrepancies are detected in the comparison of the guidelines; some of them could be attributed to the fact that (i) the guidelines were published on different dates and their recommendations are based on different data, (ii) they are based on different economic models, and (iii) they reflect different local healthcare policies. Moreover, discrepancies exist in relation to the age group of 21 to 29, both in terms of screening initiation and the screening method. More specifically, routine screening is suggested either starting from the age of 21 (USPSTF, EG), the age of 25 (ACS, ASCO, CTFPHC, CCA), or not recommended between 21 and 29 at all (the WHO). Moreover, USPSTF, CTFPHC and EG prefer cytology as the optimal method, while all other medical societies favor HPV DNA tests. Additionally, variations are observed in the recommended management strategies in cases of abnormal screening results, in women with immunodeficiency, in those with in-utero exposure to DES, and in the pregnant population. Notably, ASCO is the only medical society that considers the financial situation of each country and provides specific recommendations accordingly.

In conclusion, cervical cancer screening is essential for early detection of precancerous lesions and timely prevention of invasive cancer, particularly in low-income countries that present with the highest incidence and mortality rates. Of note, given the increasing HPV vaccination coverage in some countries, HPV-related lesions will eclipse, so the screening strategies will probably change, especially for the vaccinated populations. Therefore, the implementation of evidence-based algorithms, adapted for each setting, is important to improve health outcomes and reduce mortality, especially among high-risk or under-screened populations. Thus, well-structured screening programs would significantly reduce the global burden of cervical cancer, playing a pivotal role in advancing women’s health.

## Figures and Tables

**Table 1:** Recommendations for cervical cancer screening on average-risk women.

	USPSTF	ACS	ASCO	WHO	CTFPHC	CCA	EG
**Country**	United States	United States	United States	International	Canada	Australia	Europe
**Issued**	2018	2020	2022	2021	2013	2022	2015
**Title**	Screening for Cervical Cancer US Preventive Services Task Force Recommendation Statement	Cervical Cancer Screening for Individuals at Average Risk: 2020 Guideline Update from the American Cancer Society	Secondary Prevention of Cervical Cancer: ASCO Resource-Stratified Guideline Update	WHO Guideline for Screening and Treatment of Cervical Pre-cancer Lesions for Cervical Cancer Prevention, Second Edition	Recommendations on Screening for Cervical Cancer	Guidelines for the Management of Screen-Detected Abnormalities, Screening in Specific Populations and Investigation of Abnormal Vaginal Bleeding	European guidelines for quality assurance in cervical cancer screening
**Pages**	13	26	24	115	11	288	194
**References**	66	164	55	43	90	471	121
**Screening methods**	-hrHPV testing -Cytology -Co-testing	-hrHPV testing -Cytology -Co-testing	-HPV testing -VIA	-HPV testing -VIA -Cytology	Cytology	-hrHPV testing -Cytology -Co-testing	-hrHPV testing -Cytology
**Self-sampling**	Further research required	Not recommended (not FDA approved)	Recommended	Recommended	Not mentioned	Recommended	Recommended as an alternative
**Ages <21**	Screening not recommended	Screening not recommended	Screening not recommended	Screening not recommended	Screening not recommended	Screening not recommended	Screening not recommended
**Ages 21–29**	Cervical cytology every 3 years	**Age 21–24 years** → Screening not recommended **Age >25 years** → -hrHPV testing every 5 years Alternatively: -Co-testing every 5 years -Cervical cytology every 3 years	**Age 21–24 years **→ Screening not recommended **Age >25 years** → Maximal: HPV testing every 5 years Enhanced: screening not recommended Limited: screening not recommended Basic: Screening is not recommended	Screening not recommended	**Age 21–24 years** → Screening not recommended **Age >25 years** → Cervical cytology every 3 years	**Age 21–24 years** → Screening not recommended **Age >25 years** → -hrHPV testing every 5 years	Cervical cytology every 3 years
**Ages 30–65**	-Cervical cytology every 3 years -hrHPV testing every 5 years -Cotesting every 5 years	-hrHPV testing every 5 years Alternatively: -Cotesting every 5 years -Cervical cytology every 3 years	Maximal: HPV testing every 5 years Enhanced: HPV testing every 5 years or every 10 years after two consecutive negative results Ages 30–49 → Limited: HPV testing every 10 years Basic: HPV testing every 10 years or VIA every 5–10 years	**Age 30–50** Primary HPV testing with or without HPV 16/18 genotyping every 5–10 years Alternatively: -VIA every 3 years -Cytology every 3 years **Age 50–65** Screening may be offered in women with no screening history if feasible	Cervical cytology every 3 years	hrHPV testing every 5 years	Age 30–34 → hrHPV testing should be considered -Cervical cytology every 3 years Age 35–65 → -hrHPV testing every 5 years -Cervical cytology every 3 years
**Ages >65**	Screening not recommended If: -Adequate previous screening -Not in a high-risk group	Screening not recommended If: -Adequate previous screening -Not in a high-risk group	Maximal: individualization, screening up to 70 years Enhanced: Screening is not recommended if adequate previous screening Limited: screening not recommended Basic: screening is not recommended	Screening not recommended	**Age 65–69** Cervical cytology every 3 years **Age >70** Screening is not recommended if adequate previous screening	**Age 65–69** hrHPV testing every 5 years **Age >70–74** Screening can be stopped if adequate previous screening **Age >75** Screening at request if no previous screening	Screening not recommended
**Adequate screening**	3 consecutive negative cytology results OR 2 consecutive negative co-testing results in 10 years (last within 5 years)	3 consecutive negative cytology results OR 2 consecutive negative co-testing/hrHPV results in 10 years (last within 3–5 years)	Maximal: negative results in the last 15 years Enhanced: negative results in the last 15 years	2 consecutive negative results	3 consecutive negative cytology results in 10 years	1 negative HPV testing the last 5 years	Not mentioned
**History of hysterectomy without previous CIN2/3**	Screening not recommended	Screening not recommended	Screening not recommended	Not mentioned	Screening not recommended	Screening not recommended	Not mentioned

hrHPV—high-risk human papillomavirus, VIA—visual inspection with acetic acid, FDA—Food and Drug Administration.

**Table 2:** Recommendations on cervical cancer screening for high-risk women and specific populations.

	USPSTF	ACS	ASCO	WHO	CTFPHC	CCA	EG
**History of precancerous lesion**	Screening for at least 20 years	Screening for at least 25 years After 65 years: surveillance every 3 years	Maximal: If (+) at 60 years, screening for at least 10 years	In CIN2/3: Repeat HPV DNA in 12 months If negative, routine screening In CIN3+: HPV DNA annually for 3 years after treatment and then return to routine screening	Not mentioned	In CIN2/3: Repeat HPV DNA every 12 months If two consecutive negative results, routine screening In AIS: Screening for at least 25 years Co-testing annually for 5 years and then every 3 years	Not mentioned
**Abnormal results**	Not mentioned	Not mentioned	**Maximal:** In HPV DNA (+) → hrHPV and/or cytology In negative results → repeat HPV in 12 months In positive results → colposcopy **Enhanced:** In HPV DNA (+) → hrHPV and/or cytology In negative results → repeat HPV in 12 months In positive results → colposcopy **Limited:** In HPV DNA (+) → Cytology or hrHPV or VIA Abnormal cytology → colposcopy or VAT **Basic:** In HPV DNA (+) → VAT	In HPV DNA (+) → genotyping, colposcopy, VIA or cytology If negative → repeat HPV at 24 months (if negative, routine screening) In primary cytology (+) and colposcopy (−) → HPV DNA at 12 months (if negative, routine screening)	Not mentioned	**<50 years** HPV other (+) + cytology (−) → Repeat HPV in 12 months subsequently three times → then colposcopy **>50 years** HPV other (+) + cytology (−) → Repeat HPV in 12 months and subsequently two times → then colposcopy **If cytology (+) or HPV 16/18 (+)** → colposcopy at any age	**>35 years** Oncogenic HPV (+) → cytology If negative → repeat HPV after 12 months (if negative, routine screening) If ASC-H/HSIL → colposcopy If LSIL/ASCUS → retesting after 6–12 months or colposcopy
**No adequate previous screening**	At >65, screening may be offered	At >65, screening until criteria for cessation are met	Not mentioned	Not mentioned	At >70, screening until three consecutive negative cytology results	At >75 screening at request	Not mentioned
**Immunodeficiency**	Not mentioned	Not mentioned	Twice as many screenings as the general population	Screening at 25–49 years with -HPV DNA every 3–5 years -Cytology/VIA every 3 years In HPV DNA (+) → genotyping, colposcopy, VIA or cytology If negative → repeat HPV at 12 months (if negative, routine screening) In primary cytology (+) and colposcopy (−) → HPV DNA at 12 months (if negative, routine screening) If treated for CIN2+ → two consecutive negative HPV DNA results every 12 months before routine screening	Not mentioned	Screening every 3 years at 25–74 years If HPV (+) → colposcopy Age 20–24: consider HPV testing once Screening prior to transplantation	Biannual screening during the first year of diagnosis Annual subsequent screening If cytology abnormal → colposcopy
**DES**	Not mentioned	Pelvic examination Annual cytology Consideration of colposcopy	Not mentioned	Not mentioned	Not mentioned	Annual co-testing + colposcopy indefinitely	Not mentioned
**Vaccination**	Same screening	Same screening	Same screening	Not mentioned	Same screening	Not mentioned	Same screening
**Pregnancy**	Not mentioned	Not mentioned	Maximal: screening 6 months postpartum Enhanced: screening 6 months postpartum Limited: screening 6 months postpartum Basic: screening 6 weeks postpartum	Not mentioned	Not mentioned	Pregnancy Screening recommended Endocervical brush not recommended If HPV other (+): -cytology (−) → repeat HPV in 12 months -ASC-H/HSIL or glandular abnormalities → colposcopy If HPV 16/18 (+) → colposcopy Puerperium Screening at least after 6 weeks (ideally 3 months)	Not mentioned
**Sexual activity**	Same screening	Same screening	Not mentioned	Not mentioned	Same screening	Consider screening once under 25 in: Sexual abuse Sexually active before 14	Not mentioned
**LGBTQ**	Same screening	Same screening	Same screening	Same screening	Same screening	Same screening	Not mentioned

CIN—cervical intraepithelial neoplasia, AIS—adenocarcinoma in situ, HPV—human papillomavirus, DNA—deoxyribonucleic acid, VAT—visual assessment for treatment, DES—diethylstilbestrol, ASC-H—atypical squamous cells cannot rule out high grade squamous intra-epithelial lesion, HSIL—high-grade squamous intraepithelial lesion, LGBTQ—lesbian, gay, bisexual, transgender, queer, LSIL—low-grade squamous intraepithelial lesions, ASCUS—atypical squamous cells of undetermined significance.

## Data Availability

Data are publicly available.
